# Supplementary material for: Accurate detection and quantification of SARS-CoV-2 genomic and subgenomic mRNAs by ddPCR and meta-transcriptomics analysis
Source: Commun Biol. 2021 Oct 22;4:1215. doi: 10.1038/s42003-021-02748-0 (PMC8536764; doi:10.1038/s42003-021-02748-0)
Supplement: Supplementary file 2 — Supplementary Information [file 42003_2021_2748_MOESM2_ESM.pdf]

Accurate detection and quantification of SARS-CoV-2 genomic and subgenomic mRNAs by ddPCR and meta-transcriptomics analysis

Annarita Oranger[1+], Caterina Manzari[1+] , Matteo Chiara[2,3+], Elisabetta Notario[1], Bruno Fosso[2], Antonio Parisi[4], Angelica Bianco[4], Michela Iacobellis[5], Morena d'Avenia[5], Anna Maria D'Erchia[1,2*], Graziano Pesole[1,2*]

[1] Department of Biosciences, Biotechnology and Biopharmaceutics, University of Bari Aldo Moro, Via Orabona 4, 70126 Bari, Italy

[2] Institute of Biomembranes, Bioenergetics and Molecular Biotechnologies, National Research Council, Via Amendola 122/O, 70126 Bari, Italy

[3] Department of Biosciences, University of Milan, Via Celoria 26, 20133 Milan, Italy

[4] Istituto Zooprofilattico Sperimentale della Puglia e Basilicata, 70017 Putignano, Italy

[5] Servizio Centralizzato Aziendale di Citopatologia e Screening- PO "Di Venere" - ASL, 70131 Bari, Italy

[*] Corresponding authors

Graziano Pesole

Department of Biosciences, Biotechnology and Biopharmaceutics, University of Bari Aldo Moro, Via Orabona 4, 70126 Bari, Italy

email: graziano.pesole@uniba.it

Anna Maria D'Erchia

Department of Biosciences, Biotechnology and Biopharmaceutics, University of Bari Aldo Moro, Via Orabona 4, 70126 Bari, Italy

email: annamaria.derchia@uniba.it

[+] These authors contributed equally

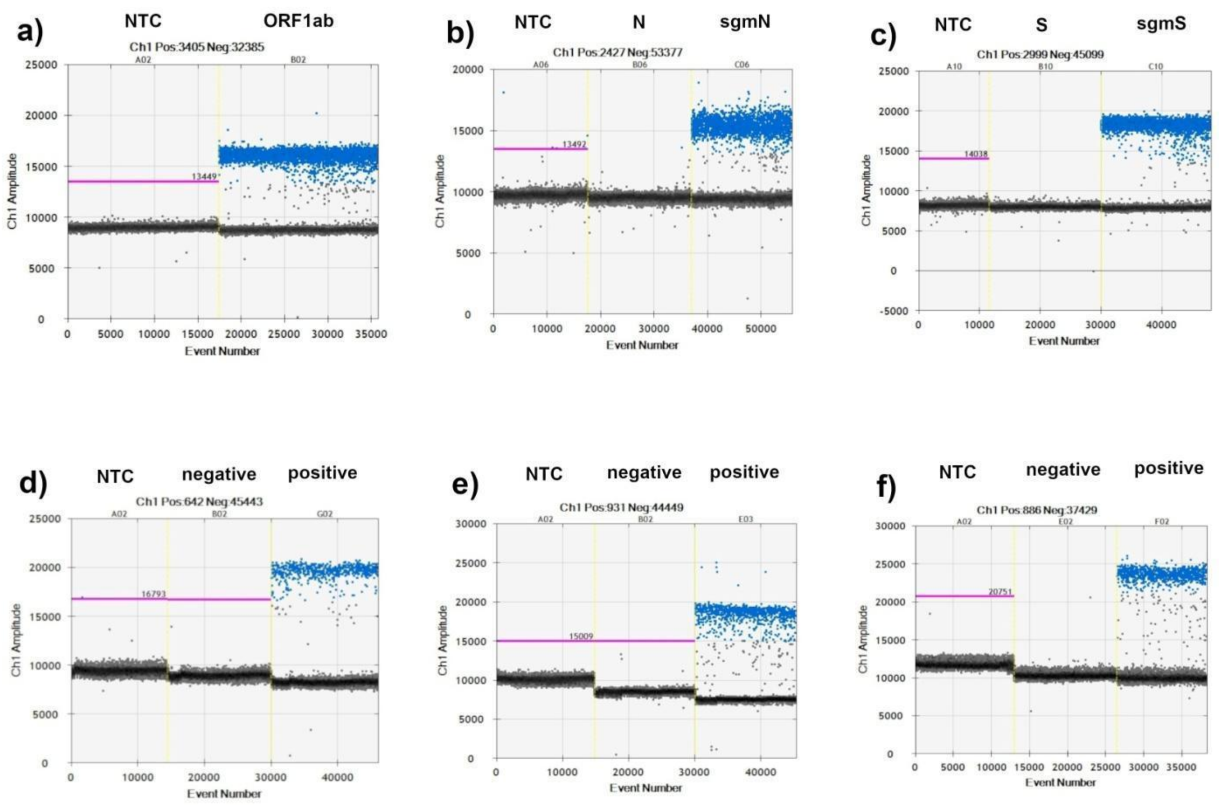

Supplementary Fig. 1. ddPCR 1D plot of SARS-CoV-2 ddPCR assays on different templates.
ddPCR 1D amplitude plots of ORF1ab (panels a and d), N sgmRNA (panels b and e), S sgmRNA (panels c and f), using as template:

a) from the left: NTC sample; custom genomic ORF1ab DNA sequence;
b) from the left: NTC sample, custom DNA sequence corresponding to N gene, custom DNA sequence corresponding to N sub-genomic mRNA;
c) from the left: NTC sample, custom DNA sequence corresponding to S gene, custom DNA sequence corresponding to S sub-genomic mRNA;
d) from the left: NTC sample; SARS-CoV-2 negative RNA sample; SARS-CoV-2 positive RNA sample;
e) from the left: NTC sample; SARS-CoV-2 negative RNA sample; SARS-CoV-2 positive RNA sample;
f) from the left: NTC sample; SARS-CoV-2 negative RNA sample; SARS-CoV-2 positive RNA sample.

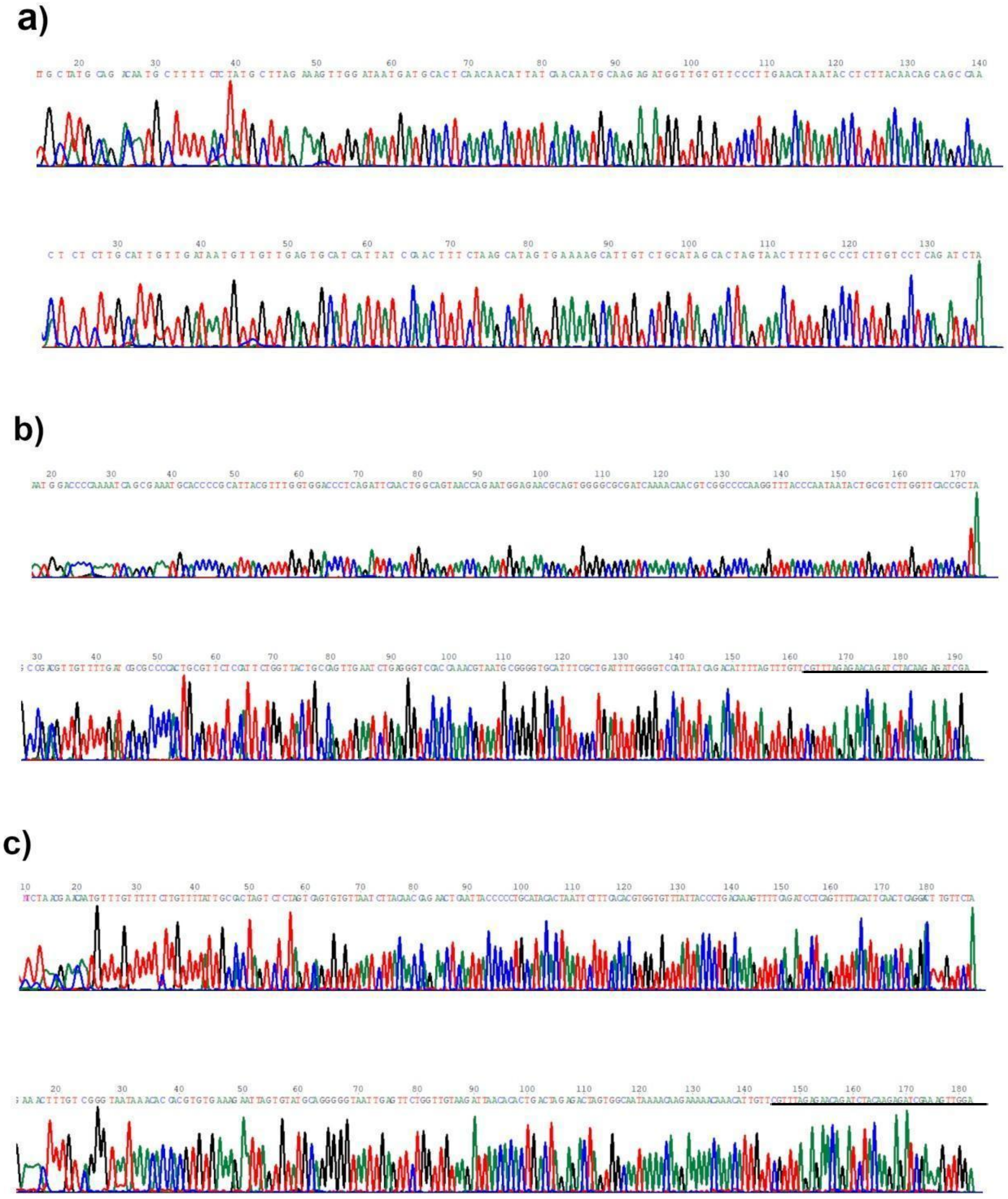

Supplementary Fig. 2. Sanger sequencing electropherograms of SARS-CoV-2 ORF1ab, sgmN and sgmS RNAs amplicons. Sanger sequencing was performed on amplicons obtained from SARS-CoV-2 positive samples; forward and reverse sequences electropherograms are shown for ORF1ab (a), sgmN (b) and sgmS (c) RNAs, respectively at the top and the bottom of each panel.

In (b) and (c), the leader sequence (LS) is underlined.

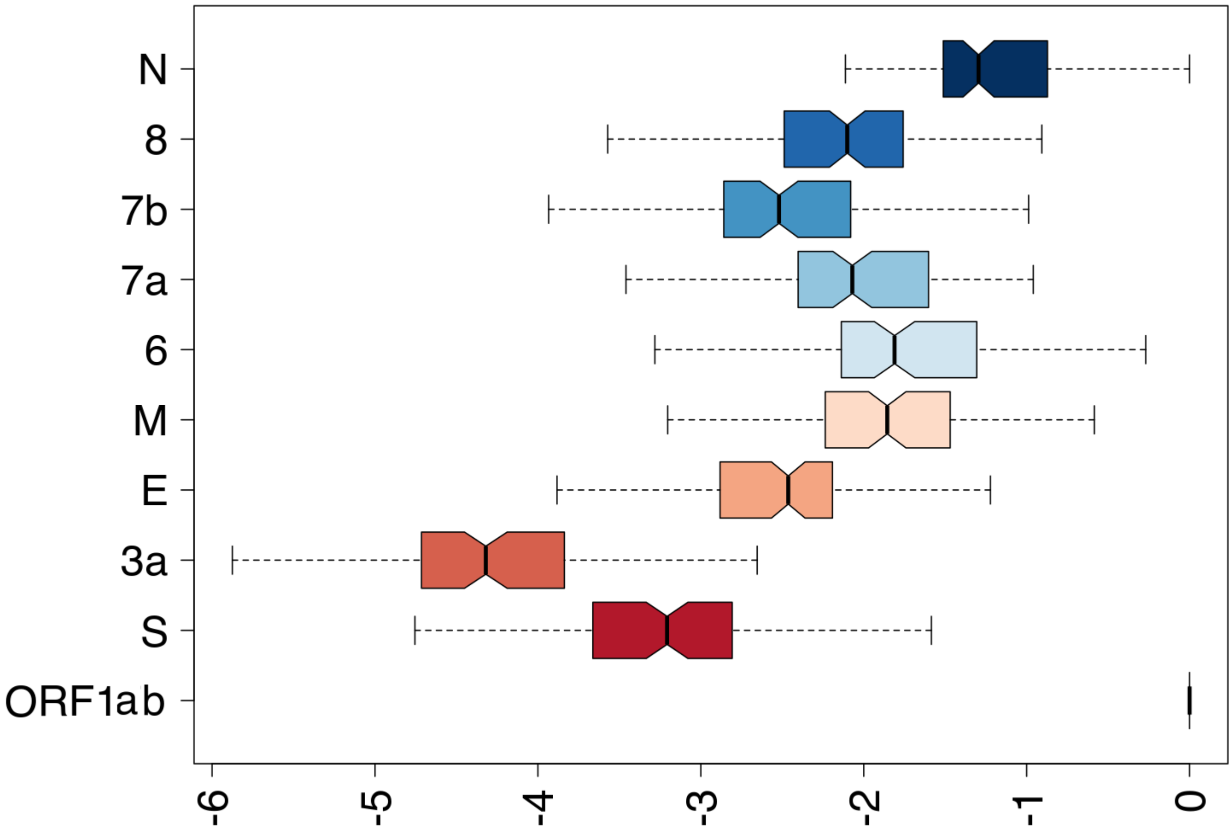

Supplementary Fig. 3. Boxplot of SARS-CoV-2 metatranscriptomic reads ratios.

Log2 ratios distributions between each sgmRNA and the ORF1ab gene are represented. Genes are indicated on the Y axis. Log ratios on the X axis.

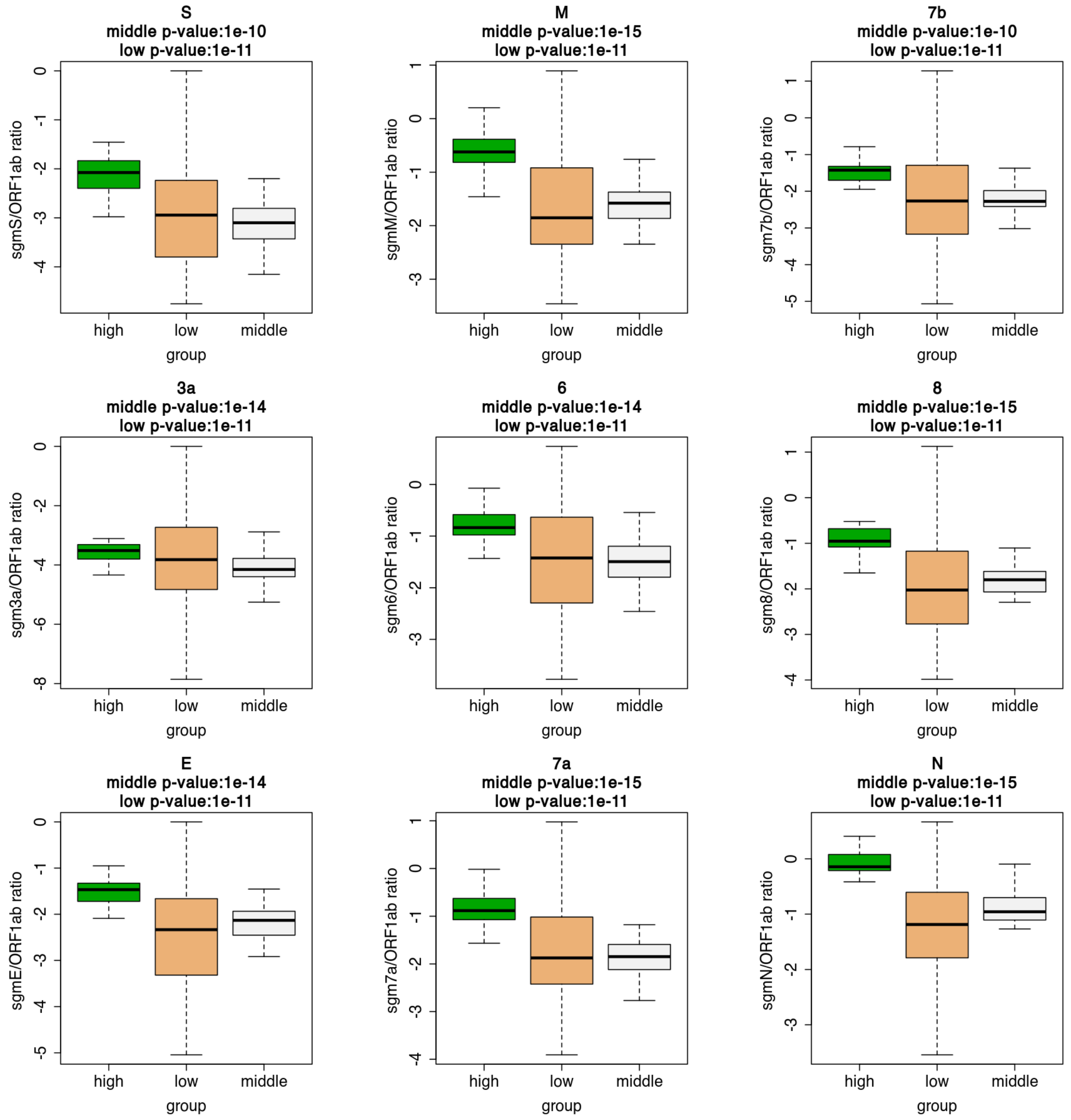

Supplementary Fig. 4. Boxplot of SARS-CoV-2 transcripts relative expression from metatranscriptomic sequencing according to viral RNA content groups.

Boxplots represent log2 scaled ratios distributions between each SARS-CoV-2 canonical sgmRNAs and the ORF1ab genomic RNA. Log ratios are represented on the Y axis; viral RNA content groups on the X axis. Significance was calculated by comparing “high” *vs* “middle+low” groups by Mann–Whitney U test / Wilcoxon rank-sum.

SARS-CoV-2 target	Sequences (5'-3')
ORF1ab	GGAAAAGATGGCTGATCAAGCTATGACCCAAATGTATAAACAGGCTAGATCTGAGGACAAGAGGGC<br>AAAAGTTACTAGTGCTATGCAGACAATGCTTTTCACTATGCTTAGAAAGTTGGATAATGATGCACT<br>CAACAACATTATCAACAATGCAAGAGATGGTTGTGTTCCCTTGAACATAATACCTCTTACAACAGC<br>AGCCAAACTAATGGTTGTCATACCAGACTATAACACATATAAAAATACGTGT
Genomic N	AGACTTTTTAGAGTATCATGACGTTCGTGTTGTTTTAGATTTCATCTAAACGAACAAACTAAAATG<br>TCTGATAATGGACCCCAAAATCAGCGAAATGCACCCCGCATTACGTTTGGTGGACCCTCAGATTCAA<br>CTGGCAGTAACCAGAATGGAGAACGCAGTGGGGCGCGATCAAAACAACGTCGGCCCCAAGGTTTAC<br>CCAATAATACTGCGTCTTGGTTCACCGCTCTCACTCAACATGGCAAGGAAGA
Genomic S	AATTAGAGAAAACAACAGAGTTGTTATTTCTAGTGATGTTCTTGTTAACAACTAAACGAACAATGT<br>TTGTTTTTCTTGTTTTATTGCCACTAGTCTCTAGTCAGTGTGTTAATCTTACAACCAGAACTCAAT<br>TACCCCCTGCATACACTAATTCTTTCACACGTGGTGTTTATTACCCTGACAAAGTTTTCAGATCCT<br>CAGTTTTACATTCAACTCAGGACTTGTTCTTACCTTTCTTTTCCAATGTTA
N sgmRNA	AGGTAACAAACCAACCAACTTTCGATCTCTTGTAGATCTGTTCTCTAAACGAACAAACTAAAATGT<br>CTGATAATGGACCCCAAAATCAGCGAAATGCACCCCGCATTACGTTTGGTGGACCCTCAGATTCAA<br>CTGGCAGTAACCAGAATGGAGAACGCAGTGGGGCGCGATCAAAACAACGTCGGCCCCAAGGTTTAC<br>CCAATAATACTGCGTCTTGGTTCACCGCTCTCACTCAACATGGCAAGGAAGA
S sgmRNA	ACCTTCCCAGGTAACAAACCAACCAACTTTCGATCTCTTGTAGATCTGTTCTCTAAACGAACAATG<br>TTTGTTTTTCTTGTTTTATTGCCACTAGTCTCTAGTCAGTGTGTTAATCTTACAACCAGAACTCAA<br>TTACCCCCTGCATACACTAATTCTTTCACACGTGGTGTTTATTACCCTGACAAAGTTTTCAGATCC<br>TCAGTTTTACATTCAACTCAGGACTTGTTCTTACCTTTCTTTTCCAATGTTA

Supplementary Table 1. Custom synthetic DNA sequences (250 bp each) used in this study.
For sgmRNAs, the portion of the Leader Sequence (LS) present in the custom DNA sequence is underlined.
